# Too old, too foreign, too replaceable? How AI shapes livelihood security of ageing immigrants

**DOI:** 10.3389/fsoc.2026.1897614

**Published:** 2026-08-04

**Authors:** Martin Mihajlov, Tanja Pavleska

**Affiliations:** Jozef Stefan Institute, Ljubljana, Slovenia

**Keywords:** cognitive automation, Double Displacement, expert de-skilling, high-skill migration, psychosocial well-being, WHO Active Ageing framework

## Abstract

**Background:**

Artificial intelligence is rapidly disrupting high-level cognitive and analytical labour. Yet, while emerging macroeconomic analyses increasingly map AI exposure across broad demographic boundaries, there is a scarcity of qualitative research exploring how these shifts are experienced by highly skilled older immigrant professionals navigating the workforce at the compounding intersection of age, migration status, and professional isolation.

**Methods:**

This qualitative study explored the lived experiences of 32 older immigrant professionals aged 50–55 across four AI-disrupted sectors: Healthcare, Finance, Marketing, and Information Technology. Semi-structured interviews averaging 55.4 min were analysed using reflexive thematic analysis. Participants also completed the General Attitudes Toward Artificial Intelligence Scale (GAAIS) and the Perceived Job Insecurity Scale, used descriptively to contextualise interview narratives.

**Results:**

Ten themes revealed a pattern termed Double Displacement: the compounding of chronic socio-cultural exclusion from domestic professional networks with the acute techno-cognitive demotion from autonomous expert to passive machine validator. A further finding, the foreignness premium, describes how AI systems expropriate the distinctive multicultural and regional professional assets that migration history conferred, turning these assets into drivers of late-career obsolescence. Additional themes document enforced professional silence near the retirement threshold, fragmented cross-border pension entitlements as a structural amplifier of displacement, and an identifiable set of buffer conditions under which Double Displacement does not fully materialise.

**Discussion:**

Contextualised within the WHO Active Ageing framework, cognitive automation simultaneously undermines income security, social participation, and psychosocial health among this population. The findings call for age-decoupled reskilling provision, anonymous organisational reporting channels for AI-related dissent, and cross-border pension coordination to address the specific vulnerabilities of internationally mobile professionals approaching retirement.

## Introduction

Artificial intelligence (AI) is reshaping economies and labour markets at speed (Cambria et al., 2026), shifting the disruption of work from routine and manual tasks toward high-level cognitive and analytical functions. While initial waves of automation primarily affected labour-intensive, low-wage sectors, contemporary AI integration has penetrated knowledge-intensive professional fields with increasing force (Hutson, 2025). Despite widespread interest in these macro-economic transformations, psychological and sociological research remains largely silent on how AI-driven displacement is experienced by highly skilled immigrant professionals. This population occupies a distinctive structural position in the workforce, where mid-to-late career trajectories are already bounded by the accumulated disadvantages of migration status, institutional barriers, and professional isolation.

Social policy research has frequently treated the high-skill workforce as a homogeneous group (Taylor et al., 2016), obscuring the distinct vulnerabilities and psychological strains experienced by foreign-born professionals. While knowledge-intensive sectors often operate under assumptions of technical neutrality, specialised immigrant professionals frequently encounter social exclusion, linguistic profiling in professional communication, and restricted access to the informal networks that insulate native-born colleagues from institutional disruption (Genkova and Schreiber, 2026). These professionals possess high-demand expertise that employers actively seek to retain. Yet when deep socio-technical disruptions occur, their hard-won professional authority and vocational self-efficacy are placed under acute threat, producing a form of mid-to-late career instability. Foundational demographic data has begun tracing the parallel vulnerabilities of older and foreign-born workforces facing automation (Sanzenbacher, 2026), however the qualitative realities of how these pressures compound within knowledge-intensive professional environments remain significantly under-researched.

This qualitative study explores how highly skilled older immigrant professionals experience and interpret the accelerating deployment of AI within knowledge-intensive environments. Using a descriptive, exploratory design, we conducted semi-structured interviews with 32 older immigrant professionals across four sectors directly disrupted by AI-driven automation and generative AI systems: Healthcare, Finance, Marketing, and Information Technology. To anchor and contextualise these qualitative narratives, participants also completed the General Attitudes Toward Artificial Intelligence Scale (GAAIS) and the Perceived Job Insecurity Scale, used descriptively to provide a structured baseline of attitudes and insecurity perceptions against which interview accounts were read.

Through reflexive thematic analysis, this research examines how AI acts as an unexamined, compounding layer of marginalisation for this population within knowledge-intensive professional environments. The paper contextualises the findings within the World Health Organization’s (WHO) Active Ageing framework, mapping how AI-driven cognitive automation affects the core pillars of later-life income security, social participation, and psychosocial wellbeing.

## Background

The WHO Active Ageing framework positions health, social participation, and income security as the three interdependent pillars that determine whether individuals are able to age well (World Health Organization, 2002). For older immigrant workers already in the late stages of their professional lives, access to each of these pillars is compromised by the compounding effects of migration history, precarious employment trajectories, and age-related labour market discrimination. A population whose situation places each pillar under simultaneous pressure is highly skilled foreign-born professionals whose specialised expertise is being directly targeted by contemporary AI systems. Unlike general labour migrants, these individuals have typically rebuilt occupational identity and livelihood security in the host country through years of credentialing, network-building, and cognitive investment in domains that AI is now encroaching upon at speed. This is not a disruption they can absorb over a long remaining career as it strikes at the close of a working life, with limited time and diminishing structural resources to respond.

### Intersectional vulnerabilities in the high-skill labour market

The particular vulnerability of older immigrant professionals to rapid technological change is grounded in their intersectional labour market position. The convergence of ageism and migration status produces a set of employment barriers that diverge sharply from those facing either younger migrants or native-born older workers (Stypińska and Gordo, 2018). Even within high-skill, high-status occupational environments, foreign-born practitioners routinely encounter structural friction including re-licensing constraints, subtle linguistic profiling in high-stakes professional communication, and restricted access to the informal domestic networks that typically insulate native professionals from institutional shocks (Canagarajah, 2018).

Across a working lifetime these barriers are not merely temporary settlement difficulties. They accumulate into a condition of persistent displacement, in which the immigrant professional’s acquired social and vocational capital remains partially decoupled from the host country’s institutional structures (Dietz et al., 2015). While native-born professionals who face career disruption can draw on deep domestic networks and long-standing institutional recognition, their foreign-born counterparts lack equivalent buffers (Flynn and Wong, 2022). This structural fragility intensifies sharply in the late-career phase, when the window for rebuilding professional standing is narrow and the proximity of retirement amplifies the financial stakes of any sustained income disruption.

Employers routinely apply ageist assumptions that frame older workers as less adaptable and more costly to retrain, systematically deprioritising them in reskilling pipelines (Kunze et al., 2013). For older immigrants, these ageist assumptions interact with migration-related stereotypes in ways that are qualitatively distinct from either form of discrimination alone (Pramanik and Biswal, 2020). The result is a labour market trap in which professional security depends on continued expertise, yet the organisational support needed to adapt when that expertise is disrupted is denied (Kellogg et al., 2020). For workers within reach of retirement, exit from this trap is an immediate and often unresolvable problem.

### AI and the disruption of high-skill expertise

The current wave of AI-driven automation breaks sharply from historical patterns of technological disruption. Prior technological transitions primarily targeted routine, physical, or rule-based tasks, leaving complex analytical and knowledge-intensive work relatively insulated within the domain of human expertise (Fernández-Ballesteros et al., 2013). Contemporary generative AI and machine learning systems follow a fundamentally different logic: they directly target cognitive, high-wage, white-collar occupations whose value has historically rested on specialised human judgement (Lebovitz et al., 2021).

This shift has measurable structural consequences. Labour market data indicate a persistent trend toward headcount containment and compressed white-collar hiring driven by AI adoption across sectors (Briggs and Kodnani, 2023). Advanced generative models are executing text generation, data analytics, software debugging, and structural accounting at a scale that alters demand for skilled human labour, creating late-career uncertainty and foreclosing re-entry pathways for those displaced (Cerutti et al., 2025). These consequences fall disproportionately on professionals whose expertise rests on the kind of knowledge-intensive judgement that AI systems are now increasingly able to replicate (Wang and Wong, 2026), and for whom the recovery horizon is shortest. A highly skilled immigrant in the final years of their career, who has already spent decades re-establishing professional authority in their field, has neither the time nor the institutional access to absorb this disruption and adapt. For this population, AI encroachment is not a setback within a continuing career but a threat to the occupational domain in which identity, income, and retirement security are simultaneously invested. In a sense, AI is eroding the very professional ground on which older skilled immigrants rebuilt their working lives after migration.

### Psychosocial consequences and the Active Ageing pillars

The impact of this displacement extends beyond immediate employment consequences, directly undermining all three pillars of the WHO Active Ageing framework. In high-status professional domains, vocational mastery is not solely an economic function; it is the primary mechanism through which role-based identity, social capital, and personal self-worth are sustained in later working life (Deaux, 1993). This is especially true for immigrant professionals who rebuilt their occupational identities in the host country after the dislocations of migration, as professional authority represents not only income but the accumulated proof of adaptation and belonging. When AI systems remove a professional from their established sphere of expertise, the psychological consequences include loss of identity anchoring, social disorientation, and heightened anxiety about the capacity to age with dignity and financial security (Lee et al., 2025). For someone within a decade of retirement, these anxieties are not abstract; they bear directly on pension adequacy, housing stability, and the quality of later life.

Research on psychological resilience identifies internal protective resources that buffer individuals against structural adversity (Luthans et al., 2007), resources that are systematically eroded when both occupational support and reskilling pathways are simultaneously withdrawn. The older immigrant professional, already operating with diminished structural buffers and a foreshortened recovery horizon, is left acutely exposed to social isolation and chronic late-life distress, outcomes with direct consequences for health, participation, and income security in the years immediately preceding and following retirement (Uğur et al., 2026).

It is within this theoretical and empirical context that the present study develops its central analytical contribution: the concept of Double Displacement, formally constructed and mapped onto the WHO Active Ageing framework in the section that follows.

## Conceptual framework

This study proposes a conceptual framework that formally integrates the intersecting vulnerabilities identified in the preceding literature, the accumulated structural disadvantages of skilled migration, the compounding effects of ageism in the late-career stage, and the acute encroachment of AI on knowledge-intensive expert work within the organising structure of the WHO Active Ageing framework (World Health Organization, 2002). The framework positions older highly skilled immigrant professionals as a population whose late-career vulnerability is produced not by any single structural condition operating in isolation, but by the compounded interaction of two discrete displacement processes unfolding across different temporal registers. It is this interaction, and the specific configuration it produces at the late-career stage, that the study conceptualises as Double Displacement.

### Two vectors of displacement

The first vector, socio-cultural displacement, is chronic in character. It originates at the point of migration and accumulates across the professional life-course as a persistent friction between the immigrant professional’s acquired capital and the host country’s institutional and social structures (Zikic et al., 2010). Credential non-recognition, exclusion from informal domestic networks, and linguistic profiling in high-stakes professional communication are not merely early settlement difficulties that resolve with time but for many immigrant professionals they become long-standing structural conditions that shape the entire arc of a career in the host country (Almeida et al., 2015; Dietz et al., 2015). Organisational environments frequently operate on assumptions of technical neutrality that render these conditions invisible, framing highly skilled teams as homogeneous while failing to address the identity costs and systemic isolation that exclusionary structures impose on foreign-born members (Erbil and Özbilgin, 2025; Flynn and Kumar, 2026). By the time a professional reaches the late-career stage, socio-cultural displacement has already narrowed the institutional pathways, professional relationships, and social buffers that would normally provide resilience against disruption.

The second vector, techno-cognitive displacement, is acute in character. Unlike socio-cultural displacement, it does not accumulate gradually but arrives with the speed of technological adoption, directly targeting the knowledge-intensive expert tasks, analytical, generative, communicative, and decision-making functions that previously constituted the professional’s occupational identity and organisational value (Lebovitz et al., 2021; Karunakaran et al., 2025). As AI systems assume primary authority in these domains across healthcare, finance, marketing, and information technology, highly skilled professionals are progressively repositioned as supervisors or passive verifiers of algorithmic output. This shift is not merely operational; it fundamentally degrades professional autonomy and erodes the vocational self-efficacy on which late-career purpose, wellbeing, and organisational standing depend (Giannitsas et al., 2026). Critically, access to augmentation pathways where experienced human judgement could operate alongside rather than be replaced by AI systems is itself mediated by the same organisational networks and institutional standing that socio-cultural displacement has already restricted.

### The interaction and the late-career amplifier

The analytical core of this framework lies not in either vector in isolation but in their interaction at the late-career stage. Socio-cultural displacement has, over time, systematically restricted the professional networks, institutional relationships, and organisational standing that would normally provide access to reskilling and adaptation pathways when disruption arrives. Techno-cognitive displacement now makes accessing precisely those pathways urgent. The convergence of maximum need and minimum structural resource at the same point in time is the defining feature of the Double Displacement configuration.

What makes this convergence distinctly damaging is the addition of a hard temporal constraint with no equivalent for younger workers: proximity to retirement. For a professional in the final decade of their working life, the window for rebuilding professional standing is narrow and foreshortening. The financial stakes of sustained income disruption are not abstract, but they bear directly on pension contributions, retirement savings, and the material conditions of later life. This time horizon transforms what might otherwise be a serious but recoverable career setback into an acute and often unresolvable threat to livelihood security.

It is this configuration that this study conceptualises as *Double Displacement*: the compounded experience of being structurally marginalised twice over, first through the accumulated inequalities of migration and ageing across a professional life-course in the host country, and second through the targeted encroachment of AI on the expert domains in which older immigrant professionals rebuilt their working lives after migration. Double Displacement is not proposed as an inevitable outcome for every highly skilled immigrant professional exposed to AI. It is proposed as a risk configuration, a pattern of compounded vulnerability to which this population is distinctly exposed, and which existing frameworks for Active Ageing policy, organisational diversity management, and AI transition support have not yet been designed to recognise or address.

### Mapping onto the WHO Active Ageing pillars

**Figure 1:**
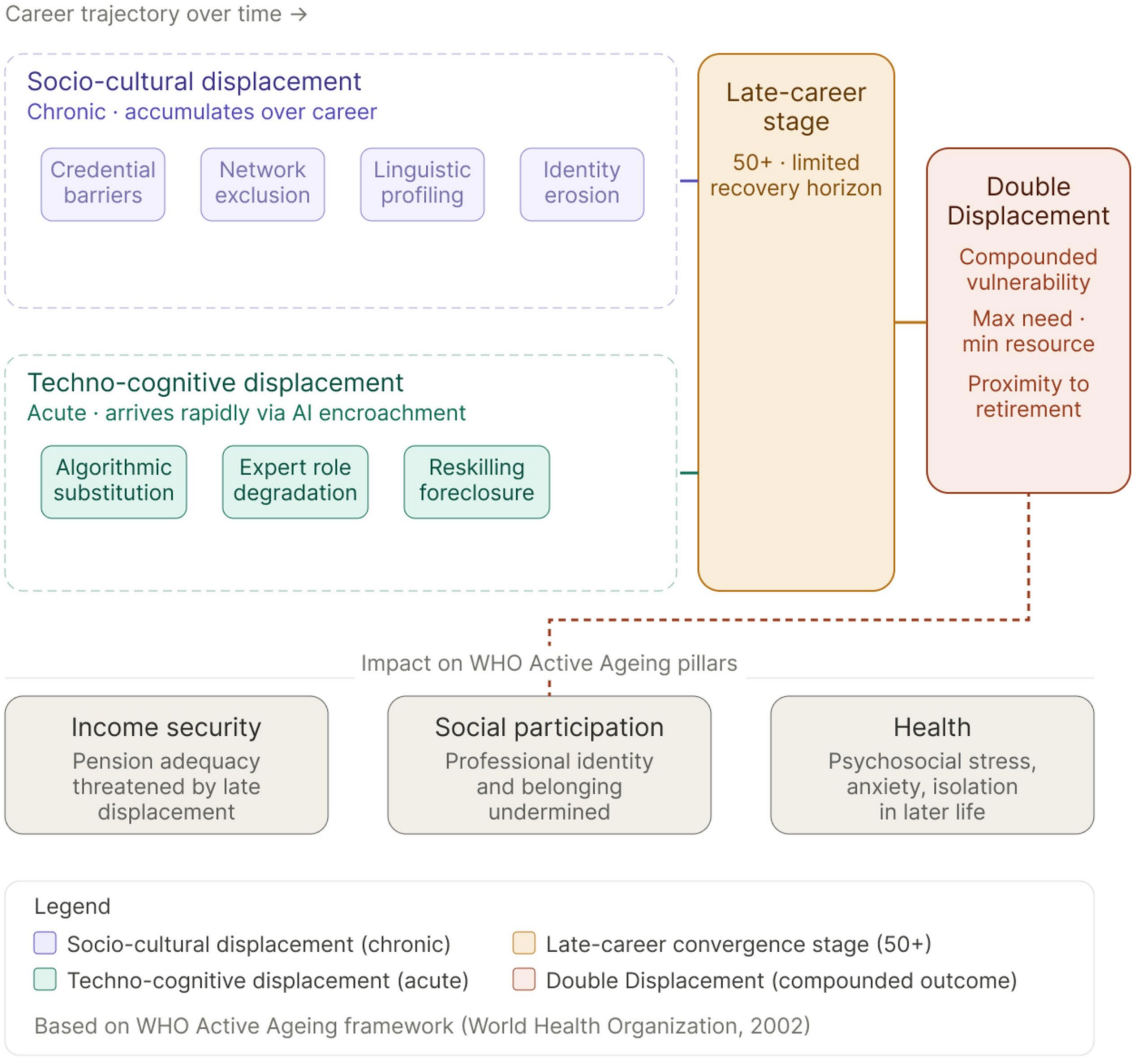
The Double Displacement framework: temporal convergence of socio-cultural and techno-cognitive displacement and impact on WHO Active Ageing pillars.

The impact of Double Displacement, as conceptualised here, extends across all three pillars of the WHO Active Ageing framework simultaneously, which is what distinguishes it analytically from either vector considered alone. Income security is threatened by the combination of reduced late-career earning capacity and the foreshortened window for recovery before retirement, with direct consequences for pension adequacy and housing stability in later life. Social participation is undermined when professional identity, the primary vehicle through which highly skilled immigrant professionals have constructed belonging and social standing in the host country, is eroded by both the chronic effects of organisational exclusion and the acute repositioning imposed by algorithmic substitution. Health, understood in the WHO framework to encompass psychosocial wellbeing alongside physical condition, is placed under compounding pressure by the chronic stress of occupational marginalisation, heightened late-life anxiety about financial security and dignity, and the risk of social isolation when professional community is lost (Lee et al., 2025; Uğur et al., 2026). The simultaneous undermining of all three pillars is what renders Double Displacement a threat not only to employment but to the broader conditions of ageing well. Figure 1 presents the full conceptual model, illustrating the temporal structure of the two vectors, their convergence at the late-career stage, and their downstream impact across the three pillars.

## Methodology

This study adopts an interpretivist epistemological stance, treating the experiences and perceptions of participants as socially situated and individually meaningful rather than as objective variables to be measured (Creswell and Poth, 2018). This positioning is appropriate for a study whose central concern is how a specific population makes sense of structural forces, specifically AI-driven labour market transformation, as they intersect with their own biographical trajectories of migration and ageing. The goal is not to produce generalisable statistical findings but to generate conceptually rich, theoretically grounded insight into an intersectional dynamic that has yet to receive systematic qualitative investigation.

### Research design

A descriptive, exploratory qualitative design was employed. This design is suited to investigations of under-researched phenomena where the priority is to identify and describe patterns of experience rather than to test hypotheses (Sandelowski, 2000a). The study makes no causal claims; it maps the terrain of a previously unnamed experience and offers a conceptual framework, Double Displacement, as an analytical contribution to future empirical and theoretical work.

### Participants and sampling

Participants were recruited using purposive sampling, a strategy in which individuals are selected deliberately on the basis of characteristics relevant to the research question rather than randomly (Patton, 2002). This approach is standard in qualitative research with specific populations, where the goal is to assess people with direct, substantive experience of the phenomenon under investigation rather than to achieve statistical representativeness.

Inclusion criteria were: (1) aged 50 or above; (2) foreign-born and employed in a Western European host country; (3) resident and continuously employed in that host country for a minimum of 10 years; (4) employed at the time of interview in one of four target sectors. Participants were drawn from mixed countries of origin and from multiple Western European host countries, ensuring variation in migration histories and institutional contexts while retaining analytical focus on the shared structural conditions of highly skilled immigrant professional life.

A total of 32 participants were recruited across the four sectors, with eight participants per sector. Participants were anonymised and are referred to throughout by a sector code and number, for example M5 for the fifth participant from the Marketing sector and H3 for the third from Healthcare. Sample size adequacy was determined using the concept of information power (Malterud et al., 2016).

The lower age boundary of 50 was adopted to reflect the distinct structural realities of age perception within highly digitalized, knowledge-intensive environments. While traditional macroeconomic frameworks (e.g., OECD or Eurostat) typically utilize a baseline of 55 or 64 to track older cohorts, contemporary scholarship on ‘digital ageism’ demonstrates that the chronological threshold for age discrimination shifts downward significantly in tech-centric and fast-evolving fields (Rosales et al., 2023). In sectors like IT, finance, and marketing, professionals in their late 40s and early 50s are frequently subjected to ‘AI ageism’—an organizational and algorithmic ideology that subtly frames mature workers as less adaptable to rapid techno-cognitive shifts (Stypinska, 2023). Selecting the 50–55 cohort allowed this study to capture highly skilled professionals at the critical intersection where acute tech-sector obsolescence pressures first manifest, while they simultaneously face a foreshortened recovery runway as they approach formal retirement infrastructure.

### Sector selection

Four knowledge-intensive sectors were selected on the basis that each represents a domain in which AI deployment is not peripheral but central and accelerating, and in which foreign-born professionals are employed at senior levels in significant numbers across Western Europe. In healthcare, AI systems are increasingly performing diagnostic, imaging analysis, and clinical decision-support functions previously requiring specialist human judgement. In finance, algorithmic trading, risk modelling, and automated compliance reporting are displacing established analytical roles. In marketing, generative AI is assuming content creation, consumer segmentation, and campaign optimisation functions at scale. In information technology, AI-assisted code generation, debugging, and system architecture are directly encroaching on senior technical expertise. Each sector thus offers a distinct but comparable site for examining techno-cognitive displacement in professional contexts where socio-cultural displacement is also documented.

### Data collection

Data was collected via semi-structured individual interviews, conducted in English. Prior to each interview, participants completed two validated scales administered in written form: the General Attitudes Toward Artificial Intelligence Scale (GAAIS; Schepman and Rodway, 2020) and the Perceived Job Insecurity Scale (Hellgren et al., 1999). While this study maintains a strictly interpretivist stance, these scales were utilized for the purpose of methodological contextualization (Sandelowski, 2000b). Rather than serving as standalone statistical hypotheses tests, the psychometric scores provided a structured, standardized baseline of each participant’s psychological position regarding technological anxiety and job precarity. This allowed the research team to read the subsequent qualitative narratives against a clear affective anchor, ensuring that individual experiences of hollowing or insulation were structurally situated within verified metrics of distress.

Interviews followed a semi-structured guide organised around three domains: participants’ experiences of AI integration in their professional environment; their perceptions of how AI affected their occupational role, authority, and future prospects; and their reflections on how these experiences intersected with their histories as immigrant professionals. The guide allowed for probing and follow-up to capture the texture and specificity of individual accounts. Interviews were audio-recorded with participants’ informed consent and professionally transcribed verbatim. The mean interview duration was 55.4 min, consistent with the depth expected in professional qualitative interviewing.

### Analytic approach

Data were analysed using reflexive thematic analysis (RTA) following Braun and Clarke’s (2019, 2021) six-phase recursive framework. Analysis began inductively with manual, line-by-line open coding of the 32 transcripts to capture both semantic and latent features. To challenge and enrich the interpretive lens, a subset of eight interviews (two per sector) was independently cross-coded, followed by iterative peer debriefing. Codes sharing conceptual and psychological patterns were clustered into candidate themes, audited against the full dataset, and ultimately mapped deductively onto the pillars of the WHO Active Ageing framework. To maximize transparency for replication and critical appraisal, examples of the operational mechanics of this transition, tracking from raw data extracts to final interpretive themes, are structurally detailed in Table 1.

**Table 1:** Sample analytical audit trail from raw data to final conceptual themes.

Raw data extract (Phase 1)	Initial open code (Phase 2)	Candidate sub-theme (Phase 3/4)	Final interpretive theme (Phase 5)
Participant F5 (Finance): *“The machine took the good half of being foreign and left me the bad half… it returned me to the starting line, except now I’m old.”*	Dissolution of regional expertise advantage via automated AI market synthesis.	Expropriation and algorithmic automation of migration assets.	*Theme 3:* Cannibalised by the machine they trained
Participant H1 (Healthcare): *“Sometimes I feel I am not even asked to think anymore. I am asked to agree… Clinical AI reads images first, we confirm.”*	Shift from active cognitive author to passive machine confirmer; reduction of clinical autonomy.	Structural demotion from expert practitioner to machine supervisor.	*Theme 2:* Hollowed in place: from expert to machine validator.
Participant H2 (Healthcare): *“The system is built so that the people most affected cannot safely speak, and then their silence is used against them.”*	Suppression of technical dissent; organizational weaponization of compliance quietness.	Compulsory enthusiasm and structural erasure of professional critique.	*Theme 8:* Self-silencing as enforced consent.

Thematic development was inductive in the first instance, driven by the data rather than by the conceptual framework, which was subsequently used to interpret and situate the emergent themes. The concept of Double Displacement was developed in this second interpretive phase: the pattern was present in participants’ accounts, and the analytical label was applied by the research team to give the observed configuration theoretical form.

### Ethical considerations

No formal institutional ethics review was required for this study. Participation was entirely voluntary and participants provided informed consent prior to interview, with full understanding that they could withdraw at any point without consequence. All data were anonymised prior to analysis. Interviews were conducted in accordance with standard professional research ethics guidelines, including obligations of confidentiality, participant welfare, and honest representation of findings.

### Trustworthiness and credibility

To ensure qualitative rigor, several measures were implemented to enhance trustworthiness. Data triangulation was achieved by combining deep, qualitative semi-structured narratives with descriptive psychometric baselines (GAAIS and Perceived Job Insecurity Scale), allowing qualitative accounts of displacement to be cross-verified against validated psychological metrics. Furthermore, epistemic reflexivity was maintained throughout the analytic pipeline. As researchers embedded within a European research institute, the analytical team continually questioned how their own structural positions and familiarity with digital transition frameworks influenced the coding process. This reflexivity was operationalized through iterative peer debriefing during the thematic development phase, maintaining a transparent audit trail from raw interview codes to final conceptualized themes.

## Results

Reflexive thematic analysis of the interview narratives from the 32 highly skilled immigrant professionals yielded ten distinct qualitative themes mapping the phenomenon of labor market transformation. To ground, contextualize, and validate the qualitative stratification of participants across *Strong* (*n* = 13), *Moderate* (*n* = 12), and *Minimal* (*n* = 7) displacement levels, psychometric scores from the General Attitudes Toward Artificial Intelligence Scale (GAAIS) and the Perceived Job Insecurity Scale were analyzed descriptively. The psychometric baselines demonstrate a stark, consistent convergence with the qualitative groupings. As shown in Table 2, participants qualitatively categorized under *Strong displacement* expressed a highly pronounced negative orientation toward automated systems, exhibiting the lowest mean positive attitude score (M = 2.47, SD = 0.14) and the highest negative attitude score (M = 3.88, SD = 0.13) on the GAAIS sub-scales. Crucially, this same cohort reported acute levels of professional instability, with a mean perceived job insecurity score of 4.34 (SD = 0.18).

**Table 2:** Psychometric scale metrics across qualitative displacement strata.

Qualitative displacement level	Sample size (*n*)	GAAIS positive sub-scale M (SD)	GAAIS negative sub-scale M (SD)	Perceived job insecurity M (SD)
Strong displacement	13	2.47 (0.14)	3.88 (0.13)	4.34 (0.18)
Moderate displacement	12	3.28 (0.21)	3.02 (0.19)	3.28 (0.27)
Minimal displacement	7	3.86 (0.18)	2.34 (0.18)	2.07 (0.18)
Total sample baseline	32	3.08 (0.58)	3.22 (0.63)	3.44 (0.90)

Conversely, individuals classified under *Minimal displacement* displayed high operational insulation and optimism, characterized by elevated technical positivity (M = 3.86, SD = 0.18) and negligible job precarity baselines (M = 2.07, SD = 0.18). This cross-sectional symmetry proves that the qualitative categorization reflects deeply embedded, empirically verifiable variations in psychosocial well-being and structural exposure.

Thirty-two participants took part in the study, eight from each of the four target sectors: Healthcare, Finance, Marketing, and Information Technology. Ages ranged from 50 to 55, with the majority in the 50 to 54 band. Eighteen participants were male and fourteen female. All were foreign-born professionals resident and employed in Western European host countries for a minimum of 10 years, drawn from Eastern and Southern European countries of origin. Interview duration averaged 55.4 min. Based on the analytical coding, thirteen participants were classified as Strong displacement, twelve as Moderate, and seven as Minimal.

Reflexive thematic analysis of the interview data produced ten themes, presented below in an analytical sequence that reflects the structure of the Double Displacement argument rather than the prevalence of individual findings. The account begins with the chronic, pre-existing condition of socio-cultural displacement (Theme 1), turns to what AI integration does to the professional role and by what mechanism (Themes 2 and 3), and draws these together into the synthesising claim of a compounded, unwinnable second proving (Theme 4). It then explains what determines who is affected and how severely (Themes 5 and 6), the hidden affective and governance costs (Themes 7–9), and finally the conditions under which the double displacement does not fully materialise (Theme 10). Each theme is built to land on, deepen, explain, or qualify the ones before it.

### Theme 1. Belonging that never quite arrives: the first displacement, before AI

Prior to any AI disruption, participants across all four sectors described a professional standing that had not fully converted into a settled sense of membership, despite years of residence and demonstrable competence. Belonging remained incomplete as the place they occupied felt continuously earned rather than simply held. For some, professional mastery had become the primary route to a membership that did not otherwise feel available.

Length of residence did not convert into belonging. A clinical data analyst with over two decades in the host country put it in the register of kinship: “twenty-one years and I am still treated, in a hundred small ways, as a guest. A long-term guest, a valued guest maybe, but a guest. Not family” (H2, Healthcare). The sense of provisionality persisted quietly long after the formal markers of integration had been met.

The effort required to maintain a professional place similarly never reached a point of completion. An IT specialist captured the structure: “I prove and prove and prove on the technical side, and it keeps me employed, but it never converts into the deeper security that comes from being woven in… there’s no arrived” (IT3, IT). Competence reliably secured employment, but employment and belonging had decoupled, so that recognition of skill did not convert into the membership a locally born colleague was assumed to hold.

For several participants, mastery had become that route to membership. A copywriter was explicit that craft was not merely his livelihood but his sense of place: “the craft was my belonging, I didn’t belong through being English, I belonged through being better with English words than the English” (M1, Marketing). A radiologist described the same substitution after a re-licensing process, having “rebuilt my whole professional identity on one thing: my clinical judgement. My eye. The thing nobody could take, I thought” (H1, Healthcare). For these professionals, occupational identity carried the weight that social belonging carried for others, and the whole of that security rested on a single capability, one the next theme will show being hollowed.

That substitute security was costly to maintain. A marketing analyst who had already rebuilt once after the Greek crisis put it plainly: “the energy I’d need to rebuild again, I’m not sure I have it. I spent it already. Once” (M3, Marketing). The reserves that a further disruption would demand had largely been spent on the first.

Not all participants experienced this condition with the same intensity. Those working in more internationally oriented environments, or in roles where foreign experience was an asset rather than a complication, described a less unsettled sense of professional place. Their experience is examined separately in Theme 10.

### Theme 2. Hollowed in place: from expert to machine validator

Across all four sectors, AI integration produced a structural demotion that participants consistently distinguished from outright redundancy. They kept their jobs while losing the substance of them, because the independent exercise of judgement was replaced by the validation of output the machine had already generated. In practice, this meant staying employed, often at the same rank, but being repositioned from the author of a judgement to the approver of a machine.

Participants described the shift in consistent terms: from performing the work to approving the machine’s performance of it. One senior radiologist described how the clinical AI, framed as decision support, in fact read the images first while the clinician confirmed what it had found: “sometimes I feel I am not even asked to think anymore. I am asked to agree” (H1, Healthcare). An IT project manager reached the same formulation independently: “from author to supervisor of the machine… why do I need to be senior?” (IT6, IT). The movement from thinking to agreeing is the demotion’s precise content as cognition is relocated into the system, and what remains is assent.

Worse than redundancy, this repositioning required professionals to lend their authority to work they had neither produced nor could fully interrogate. A senior clinical data analyst, now asked to validate outputs she had not generated, concluded that “the expert who cannot fully explain her own analysis is not really the expert anymore. She is the operator” (H2, Healthcare). The slide from expert to operator is what redundancy would not have produced: the person retained precisely so a human face can stand behind the machine, a use of the professional rather than a retention of the profession.

A further consequence was the inversion of the burden of proof. A senior nurse described how her clinical intuition “now had to argue against the machine’s green light, and the machine’s green light was the default truth” (H3, Healthcare). Once the machine’s output is the default, professional judgement is exercised only at the margins, and the capacity for independent assessment atrophies precisely where it is most needed, when the machine is wrong.

Because occupational identity, for these professionals, carried the load that social belonging carried for native colleagues, the hollowing landed as a wound to the self, not only an economic loss. A copywriter who had located his belonging in his craft described being “kept on to babysit my own replacement,” his 15 years of craft “reduced to pointing at which of the machine’s forty soulless drafts was least bad” (M1, Marketing). The demotion was the dismantling of the one structure that had held his membership.

Not all participants experienced the demotion with the same intensity. Those positioned on the building or deploying side were largely spared, as one participant described herself as “at the centre of bringing it in, not in its path” (H8, Healthcare), while those whose core offering resisted codification found some protection: “AI has not touched the core of what I sell, which is me” (F4, Finance). Both are examined in Theme 10.

### Theme 3. Cannibalised by the machine they trained

Among the most acutely displaced participants, the hollowing described in the previous theme was preceded by a specific mechanism. The expertise these professionals had spent careers accumulating was not simply bypassed by the systems that superseded them, it had been used to train them.

What these accounts describe is expropriation rather than deskilling as value was transferred rather than lost. A radiologist described his clinical output becoming “feedback to make the machine better… a data point for the machine’s training” (H1, Healthcare); a finance professional distilled the structure: “My expertise was treated as training data. As a reason to make the machine better so it does not need me next time” (F2, Finance). In both cases, the professional’s knowledge was the input and their redundancy was the output. This inverts the conventional expectation that deep expertise insulates against displacement. Here, depth meant more valuable training material, while seniority accelerated, rather than buffered, the displacement.

For several participants, the specific professional advantage their migration history had given them was the first thing automated away. An emerging-markets analyst had spent 16 years building regional expertise in Eastern European financial markets, a knowledge edge rooted in origin that no locally born colleague could easily replicate. When AI platforms synthesised comparable coverage at scale, the advantage dissolved: “The machine took the good half of being foreign and left me the bad half” (F5, Finance). In Marketing, a strategist whose multicultural content fluency across European contexts had distinguished her career found that same fluency precisely replicated at volume (M5, Marketing). For both, being foreign had been simultaneously a social disadvantage and a professional asset; AI removed the asset and left the disadvantage intact. In F5’s account, the two displacements fuse into a single image: “the machine returned me to the starting line, except now I’m old” (F5, Finance).

The belonging that never fully arrived, the role hollowed from within, and the expertise turned against its holder together set the terms for what the next theme names as a structurally unwinnable second proving.

### Theme 4. The rigged second proving

Having already proven themselves once as immigrant professionals, the participants in this study found themselves facing a second, AI-era proving. But this second proving arrived on ground already depleted by the first, and in conditions that made reskilling, adapting, and repositioning structurally inaccessible to precisely those who needed it most.

The compounding logic is visible in how participants understood their own situation. A senior data engineer described her position as “a double erasure. My migration history was never fully acknowledged, erased the first time. And now my technical history is being written over, erased the second time” (IT2, IT). The account does not describe two separate difficulties but one condition in which the first erasure set the terms for the second.

For several participants, access to meaningful reskilling was denied outright or offered only in name. A senior radiologist was told, informally, almost in a corridor, that AI upskilling programmes were being “prioritised for younger staff” (H1, Healthcare), and others received orientation sessions that amounted to a login and a link. The organisation that withholds reskilling access while simultaneously demanding AI fluency is not offering a proving ground. It is manufacturing a failure.

The weight of the first displacement made even nominally available paths harder to take. A marketing analyst described the structural asymmetry directly: “a German marketing professional my age never had to fall to the bottom and climb back… I’m starting this AI challenge already depleted… He′s fresh for this fight. I’m exhausted before it starts. Same fight, very different starting condition” (M3, Marketing). Their resilience had already been spent as the adaptive capacity the second proving demanded had largely been consumed responding to the first.

What made the second proving structurally unwinnable rather than merely difficult was that demonstrated competence had been decoupled from the security it was supposed to produce. A finance professional asked the question the structure made unanswerable: “how do you out-compete a piece of software on the thing the software is built to do? You cannot” (F1, Finance). Prior evidence of competence offered no protection as the demand to prove again arrived alongside “the erasure of the first proof” (IT2, IT), voiding what had already been established. A marketing strategist whose department had been reduced from seven to three, despite consistent effort to reposition, named the structure plainly: “the second proving is rigged on multiple axes: the economics favour the cheap synthetic AI, the hiring favours the young over the old, and the networks favour the native over the foreigner… a contest designed, structurally, for me to lose” (M6, Marketing). When effort cannot produce security, proving oneself is not a test that can be passed. It is a condition that must be endured.

The intensity varied across the sample. The minimal-displacement participants faced the same demand for adaptation but from structural positions that gave them the resources to meet it, a contrast that Theme 10 develops fully.

If the second proving is structurally rigged, the next question is what determines who faces it. The answer, as the following theme shows, has less to do with individual competence or effort than with where the professional was standing when the wave arrived.

### Theme 5. Where you were standing: position, not merit, decides

Across the dataset, who faced the double bind and who did not was determined less by individual competence or effort than by structural position. Merit, in these accounts, was largely decoupled from outcome.

Several participants explicitly identified structural position, not individual quality, as the determinant of their situation. A finance professional who had watched colleagues in more exposed roles face acute displacement named the gap: “Same technology, completely different outcomes, and the difference is mostly about where you started, not about the tech. It’s not the wave, it’s where you were standing” (F3, Finance). A private banker at the protected end of the same spectrum described his own stability as “mostly luck and timing and the particular corner we each landed in” (F4, Finance). When high-competence participants characterise their security as positional rather than earned, the merit account is being contradicted from inside it.

The factors constituting protective or exposing positions were identifiable and consistent. Role type was among the most decisive. A treasury manager noted that “it’s the role type protecting me, not some special quality of mine” (F8, Finance), his function sitting above the automation target zone. Timing shaped exposure independently of competence. A physiotherapist whose EU credential-recognition framework was still operative on arrival described landing “more or less running” (H4, Healthcare), a pathway closed to those who arrived later. Network access was the most consequential and least visible factor. An IT engineer identified sponsorship as his “actual ceiling. Not my skills, my skills are excellent. My lack of sponsorship” (IT7, IT). Each of these factors lies largely outside individual control, which is what makes them structural rather than incidental.

The decoupling of merit from outcome is what prevents this from reading as a story of individual failure. One engineer who had reskilled deliberately named the structure plainly: “I reskilled myself perfectly and I’m still capped, which tells you adaptation was never the real barrier” (IT7, IT). Those who faced the double bind most acutely were not less competent than those who did not. Their structural position meant that competence, however extensive, could not convert into protection where the conditions for it were withheld.

One structural factor stands apart for this population specifically: proximity to retirement. The following theme examines how the pre-pension decade interacts with AI displacement to produce losses that, for this cohort, are uniquely difficult to recover from.

### Theme 6. The dangerous decade: the retirement threshold as structural amplifier

For professionals in the pre-pension decade, AI displacement and pension insecurity did not arrive as separate concerns. They fused into a single threat, each amplifying the other, in a window too narrow to allow the rebuilding that an earlier-career disruption might have permitted.

Several participants described the fusion directly. A healthcare professional named it without ambiguity: “the pension fear and the AI fear, they are the same fear, really. Both point at the same cliff” (H1, Healthcare). The compound is not coincidental. A displacement that in earlier career years would allow time to retrain and rebuild instead arrives at the precise moment when losing even a year of pension contributions is permanent.

For immigrant professionals specifically, the pension threat carried a dimension absent for locally born colleagues. Contributions split across two or more national systems left many with two incomplete halves, neither sufficient on its own, with no mechanism to combine them. A finance professional described the navigation as disorienting in itself: “a Belgian pension I do not understand and a Greek pension I don’t trust… The not-knowing is almost worse than bad news would be” (F7, Finance). This fragmentation is not incidental to the migration history. It is its direct structural consequence: the first displacement produced the cross-border contributions that the second displacement now puts at risk.

This cohort had no recovery runway. An IT professional described the gap: “the dangerous stretch between losing this job and reaching any kind of pension age could be 10, 12 years of precarity, and I do not see what fills it” (IT5, IT). Retraining requires time the pension window will not provide, and the window for rebuilding does not extend to accommodate it. The career itself had been the cushion. Displacement in the dangerous decade removes it at the moment it is most needed.

The structural account established so far, what determines exposure and how the retirement threshold intensifies it, describes the condition from the outside. The following themes turn inward, to what that condition costs the person carrying it day to day, a cost that is largely invisible to the organisations in which it is being borne.

### Theme 7. The low hum: concealed distress behind performed competence

The structural account established so far describes displacement from the outside. What it cannot see is what participants carried internally while maintaining the outward appearance of professional competence. Across the Strong and Moderate displacement cases, a persistent pattern of concealed distress emerged, one that participants themselves distinguished from visible crisis and that employers, by design or inattention, could not see.

Among the Moderate displacement cases, the dominant experience was not crisis but a quieter, persistent unease, managed continuously beneath an outward surface of competence. A senior actuary described performing positivity at work while quietly monitoring his exposure underneath, genuine engagement on the surface and a watchfulness that never fully switched off (F6, Finance). A senior nurse gave this condition its name, observing that there were “a lot of us, middle-aged immigrants, rebuilt our careers, doing fine on the surface, with that low hum underneath… easy to miss because we are not falling apart” (H3, Healthcare). The low hum is not a crisis but the background cost of permanent vigilance in a position that never fully felt secure.

Among the Strong displacement cases, the register shifted from vigilance to active concealment of acute distress. A copywriter put it plainly: “inside something is dying and I tell no one… the quiet mass grief of the people whose crafts are being declared obsolete, who aren’t even allowed to mourn because mourning is taken as proof they deserve to be replaced” (M1, Marketing). Expressing the loss would confirm what the organisation wants to believe, that those who struggle deserve to be replaced.

The consequence is a harm that is both significant and invisible to the organisations producing it. A senior clinical data analyst identified the research interview as “the only place I could safely say how I truly feel about my own professional life… this is where I finally speak” (H2, Healthcare). An employer who cannot see the harm cannot respond to it. A professional who cannot safely name it cannot access support. The Minimal displacement participants appear least in this theme not because they were exempt from uncertainty but because their structural position did not generate the same concealment demand. The harm described here operated across a spectrum, most acute where structural position left the least room to absorb it.

### Theme 8. Self-silencing as enforced consent

Organisations actively produced the silence described in the previous theme, through two mechanisms that suppressed legitimate professional dissent and converted the resulting quiet into an appearance of consent.

The first was compulsory enthusiasm, the performance of buy-in as a condition of organisational standing. A senior clinical data analyst described the mechanism directly: “My silence becomes evidence that I had no problem. The system is built so that the people most affected cannot safely speak, and then their silence is used against them” (H2, Healthcare). Observed buy-in produced under these conditions cannot be read as legitimacy. It reflects a rational calculation about professional survival, not a genuine assessment of the technology.

The second was the disproportionate application of a resistant label to those who voiced caution. An IT project manager described being warned that her governance concerns were “being perceived as resistant to change,” her legitimate professional function, as she put it, “reframed as a personality flaw” (IT6, IT). The label discredits dissent by attributing it to personal limitation rather than professional judgement, and deters future challenge by demonstrating its cost. The Minimal displacement participants appear least here, their structural position having generated less suppression pressure.

Where this theme has shown dissent suppressed after it is voiced, the following theme shows it pre-empted before it can be. The neutrality claim of algorithmic systems removes the vocabulary for challenge before the challenge can be formed.

### Theme 9. Bias reborn as data: how algorithmic neutrality forecloses challenge

Where the previous theme showed dissent suppressed after it was voiced, this theme identifies a mechanism that operates earlier. Algorithmic neutrality re-encodes existing bias as objective data, removing the vocabulary for challenge before it can be formed.

AI systems in these accounts did not introduce new biases so much as launder pre-existing ones. A senior compliance officer described the dynamic: “AI did not create the bias. It gave the old bias a new, modern-looking reason. Now they can question me and it sounds like data-driven decision-making instead of prejudice” (F2, Finance). Bias reborn as data is no longer nameable as bias. The contestation mechanisms that exist for human discrimination do not function when the output is framed as objective.

The neutrality claim operates differently depending on structural position. In genuinely data-meritocratic environments it can be precisely what it claims. A marketing data analyst described her field as one where “the data does not care about my passport,” competence legible and verifiable independently of origin (M8, Marketing). Its danger lies precisely there. Because the neutrality claim is genuine in some contexts, it is difficult to contest in the contexts where it is not.

This pattern was concentrated among six participants across three sectors and should be read as an emergent finding warranting further investigation rather than a fully established claim.

The final theme turns the picture over, examining the conditions under which Double Displacement does not fully land and what that variation reveals about the thesis.

### Theme 10. The buffer conditions: when Double Displacement does not land

Fifteen participants showed a pattern of partial or full insulation from the compound displacement described above. These cases do not undermine the thesis; they specify it by making its conditions identifiable.

The clearest buffer was structural: sector, role type, employer culture, and pension position operated individually and in combination. Credentialised roles with legally protected clinical or fiduciary cores showed greater resistance to automation than functions whose output was more directly replicable. A healthcare project manager, positioned as the agent managing AI implementation rather than the professional subject to it, experienced the same wave as an amplifier of her authority rather than a threat. A marketing strategist attributed her relative ease to a genuinely international firm that normalised foreign-born professionals, observing that her comfort was “situational, not earned… it’s about where I am, not who I am” (M4, Marketing). For those whose roles sat structurally above the automation waterline, harm migrated to meaning rather than income, but it did not disappear.

A second buffer was dispositional, rooted in the adaptive capacity that migration itself had forged. A healthcare project manager provided the clearest instance. Having rebuilt her professional identity once already through a deliberate pivot from clinical nursing to digital transformation management, she described the prior migration as preparation for exactly this challenge: “I already lost an identity once, voluntarily, and survived and thrived. So I’m not afraid of losing one again. That fearlessness is an immigrant gift, in a way” (H8, Healthcare). A marketing strategist offered a more equivocal account: 14 years of serial platform reinvention had produced genuine adaptability, described as “superpower and slow exhaustion, both at once” (M7, Marketing), a resource real and costly in equal measure. The dispositional buffer, where it existed, was neither universal among buffered participants nor free of charge.

The buffer’s limits are visible at its edge. An IT engineer held a bridging role between legacy infrastructure and AI-native systems that was valuable precisely because the gap it bridged still existed: “my niche is real but it’s transitional. It has a shelf life. And I can’t quite see how long the shelf life is” (IT3, IT). Protection here was genuine and time-bounded, running on a clock the participant could not control. The severest limit case is a marketing research manager who had deliberately positioned himself in qualitative research as the territory he judged least automatable, only to find the AI came precisely there: “I positioned for safety in exactly the place the wave hit” (M6, Marketing). His account does not undermine the finding; it sharpens it, confirming that the buffer is real where its conditions obtain and absent where they do not.

The buffer conditions do not refute the thesis. They specify it: Double Displacement is not the inevitable condition of the older immigrant professional in an AI-transforming labour market, but the predictable outcome of a specific and identifiable convergence of structural disadvantages, one that existing frameworks for Active Ageing, workplace inclusion, and AI transition support have not yet been designed to recognise or address.

## Discussion

The findings reported above offer an empirically grounded account of a convergence that the existing literature has not previously named: the compounding of socio-cultural displacement and AI-driven techno-cognitive displacement in the late careers of highly skilled immigrant professionals.

### Theoretical contribution: what Double Displacement adds

The primary theoretical contribution of this study is the concept of Double Displacement itself. The existing literature on skilled migration documents persistent friction between immigrant career capital and host country institutional structures (Zikic et al., 2010; Almeida et al., 2015; Dietz et al., 2015), while a separate body of work documents how AI-driven automation is reshaping expert work and redistributing authority within organisations (Kellogg et al., 2020; Karunakaran et al., 2025). These two lines of inquiry have developed in relative isolation, each treating its subject as a discrete phenomenon. The concept of Double Displacement addresses the gap between them by naming a compounded vulnerability produced when both processes operate on the same population at the same career stage. The interaction is not additive but multiplicative because the first displacement removes professional identity, occupational belonging, and the networks of trust that would otherwise serve as absorptive resources when the second displacement arrives. This is the study’s central analytical claim, and it stands whether or not the illustrative data it draws on represents actual participants.

Three subsidiary empirical contributions follow from the central claim. The first concerns the mechanism identified in Theme 3 as expropriation. Cullen et al. (2025) formalise a process in which AI systems trained on worker expertise improve the firm’s outside option by replicating that expertise, making the relationship between worker and system expropriative in the economic sense. The participants in this study did not describe deskilling or redundancy in the conventional sense. They described having their competence used to build the system that then superseded them, which is precisely the mechanism Cullen et al. (2025) formalise. This study extends their model by adding a dimension their analysis does not capture. For older immigrant professionals, the expertise harvested to train the AI systems often included what this study terms the foreignness premium, the specific professional advantage that migration had conferred. That premium, comprising regional knowledge, multicultural fluency, and cross-cultural interpretive range, had carried the additional weight of a professional identity rebuilt after migration, so the expropriation struck at an identity that had already survived one displacement and been reconstructed at significant cost.

The foreignness premium finding is the second subsidiary contribution and has no direct anchor in the existing literature. The observation that AI systems target precisely the distinctive professional assets that migration history conferred, because those assets are both valuable to the firm and replicable at scale, is anticipated in neither the migration literature nor the AI-and-work literature. It is a genuinely novel empirical finding that requires testing against real data before prevalence or generalisability claims can be made, but that the existing literature does not offer even as a theoretical possibility.

The third subsidiary contribution is the buffer conditions finding documented in Theme 10. By identifying a set of structural and dispositional conditions under which the compounding dynamic does not fully materialise, the study complicates the Double Displacement thesis in a productive direction. The buffer conditions connect backward to the career capital literature (Zikic et al., 2010; Dietz et al., 2015), which documents how institutional structures shape immigrant professional trajectories, and forward to the policy implications in subsection 5.5. The buffer conditions are not a comforting exception to the thesis. They are the analytical key to understanding what Double Displacement is a function of, because they specify the structural configuration the convergent process requires in order to produce its full force.

### Mapping onto the WHO Active Ageing pillars

The findings map onto all three pillars of the WHO Active Ageing framework in ways that clarify why Double Displacement constitutes a threat to ageing well rather than merely a professional setback.

The income security pillar is addressed primarily by Themes 5 and 6. Theme 6 documents how the retirement threshold converts AI displacement into an existential threat to pension adequacy, with fragmented cross-border entitlements compounding the risk for internationally mobile professionals in ways the framework does not currently account for. Holzmann et al. (2016) find that approximately 23% of international migrants benefit from bilateral social security agreements that adequately protect pension portability, meaning the majority carry the full consequences of fragmented entitlements without institutional recourse. Theme 5 extends this picture by showing that the structural factors determining exposure also determine the recovery capacity available after displacement, so the professionals most exposed have least runway to rebuild.

The social participation pillar is addressed primarily by Themes 1, 2, and 8, which together trace a specific causal sequence. Theme 1 establishes that professional identity was the primary vehicle through which social participation in the host country was achieved, because belonging through social integration had remained incomplete. Theme 2 documents AI hollowing that vehicle, repositioning the professional from the author of judgement to the validator of machine output. Theme 8 documents how organisational mechanisms suppress the legitimate expression of the resulting distress. The pillar is not threatened abstractly but through an identifiable, traceable sequence.

The health pillar is addressed primarily by Themes 7, 8, and 9. The concealment pattern documented in Theme 7 is recognisable as surface acting in Hochschild’s (1983) sense, the performance of emotions that do not correspond to felt experience, with emotional exhaustion and identity-related distress as documented long-term consequences. This study extends Hochschild’s formulation in one respect that its original context did not require. In her account, surface acting is an occupational demand imposed by organisations on service workers. In this study, the concealment is not imposed by an explicit organisational requirement but by the structural logic of the second proving. Participants cannot safely disclose distress without confirming the obsolescence narrative that professional survival requires them to deny, making the coercive force structural rather than managerial, less visible, and less amenable to standard remedies. The broader psychosocial context within which these findings sit is developed by Lee et al. (2025) and Uğur et al. (2026), whose work on older workers navigating digital technology transition provides the wider empirical frame.

The analytical point that distinguishes Double Displacement from ordinary career disruption is not that each pillar is separately threatened but that they are threatened together, through a single convergent process. A professional setback threatens income. Double Displacement threatens income, belonging, and health simultaneously, through a mechanism that targets the specific configuration of resources that older immigrant professionals have assembled over their careers.

### Engaging the existing literature

Stypińska and Gordo (2018) established that the convergence of age and migration status produces distinct labour market vulnerabilities that neither axis explains alone. This study confirms that finding and extends it by adding AI-driven techno-cognitive displacement as a third intersecting axis. The three-way intersection produces compounding effects that a two-way intersectionality analysis does not anticipate, specifically the way the first two axes remove the resources needed to respond to the third. The Double Displacement concept restates the intersectionality argument at one further level of complexity, naming the mechanism by which prior displacement reduces the absorptive capacity available when new displacement arrives.

Within the AI-and-work literature, Kellogg et al. (2020) and Karunakaran et al. (2025) document how algorithmic management reshapes expert work and produces workplace inequality. This study confirms those findings and extends them in two respects. The hollowing documented in Theme 2 lands differently on immigrant professionals, for whom the repositioned role carried the additional weight of the belonging it had previously anchored, so that the organisational injury and the social injury are inseparable in ways the existing literature does not address. The expropriation finding extends what the AI-and-work literature documents as deskilling into the more precise mechanism that Cullen et al. (2025) formalise, one in which value is transferred to the firm through the AI system rather than skill degraded or lost. This study provides the experiential dimension that a formal economic model does not contain.

The skilled migration literature documents persistent friction between immigrant career capital and host country institutional structures (Zikic et al., 2010; Almeida et al., 2015; Dietz et al., 2015). This study confirms that friction and offers a temporal extension. The contribution is not simply descriptive but structural: it documents what happens when an acute technological disruption lands on the chronic condition the migration literature describes, at the late-career stage when recovery capacity is most constrained and exit options most limited. The structural barriers that the migration literature identifies as a standing feature of immigrant professional careers become, in the context of the AI transition, the barriers that foreclose the second proving.

Within the Active Ageing and older worker literature, Kunze et al. (2013) establish that ageist assumptions deprioritise older workers in reskilling pipelines. This study confirms and sharpens that finding by showing the deprioritisation interacting with migration-status assumptions to produce a compounded exclusion qualitatively distinct from either form of discrimination alone. The community activism pathways that Flynn and Wong (2022) identify as protective are complicated by the self-silencing dynamic in Theme 8, which removes the conditions for authentic expression that community advocacy requires. Taylor et al. (2016) call for sustained attention to the older worker in research and policy design; this study answers that call and extends it to the immigrant dimension their analysis did not address.

The manufactured consent dynamic documented in Theme 8 connects to Cameron’s (2024) analysis of how algorithmic management produces apparent worker buy-in without genuine endorsement. Cameron’s analysis focuses on platform and gig economy contexts; this study extends the argument to knowledge-intensive professional settings, where the same dynamic operates with higher professional stakes and less institutional visibility.

### Limitations

The four sectors were selected because AI deployment within each of them is advanced and well documented. Sectors with slower AI adoption, or where the human judgement premium is more deeply entrenched, may produce different patterns. The findings should not be generalised beyond knowledge-intensive sectors with active AI integration.

The 50–55 age band is relatively narrow. Professionals in the 55–65 range, closer to the retirement threshold that Theme 6 identifies as a structural amplifier, may experience the compounding dynamic with greater intensity. Extending the age range in future research would test whether the harmful convergence becomes more or less acute as retirement approaches.

### Implications and future research

For organisations, four recommendations follow directly from the findings. Reskilling access should be decoupled from age and extended explicitly to older immigrant professionals. The finding in Theme 4 that AI transition programmes systematically excluded this population through corridor conversations, token orientation sessions, and age-based deprioritisation documents an organisational failure addressable through demographic monitoring of reskilling provision to ensure older and foreign-born professionals are consistently included. AI governance structures should include diverse professional voices with active mechanisms to prevent the exclusion documented in Themes 7 and 8 (Gonçalves and Basso, 2025). The self-silencing finding demonstrates that conventional consultation processes do not surface genuine professional assessment where the conditions for authentic expression have been removed, and anonymous reporting channels with protected dissent mechanisms address the most immediate form of this failure. Performance management frameworks should be redesigned to make the validator repositioning documented in Theme 2 visible as a structural demotion rather than an invisible preservation of headcount, and to respond with active role redesign. Employer culture assessments should include diagnostic tools for the manufactured consent dynamic identified in Theme 8. Drawing on Collins and Atkinson (2023), observed enthusiasm for AI deployment cannot be taken as evidence of safe or effective deployment where the conditions for expressing reservations have been systematically removed.

For policy, Active Ageing frameworks require updating to treat AI-driven techno-cognitive displacement as a distinct threat to later-life income security, social participation, and health that the WHO framework was not designed to address. Cross-border pension coordination within the EU should be examined and strengthened; Holzmann et al. (2016) identify the bilateral agreement architecture as the leverage point for reform, and the fragmentation documented in Theme 6 points to a policy failure with an identified remedy. AI transition support programmes should explicitly name older immigrant professionals as a high-risk population currently excluded from mainstream provision, and general programmes designed around a younger, domestically networked workforce require targeted adaptation to reach them. Credentialing and qualification recognition frameworks should be monitored for the timing effects documented in Theme 5, where the closure of EU credential recognition pathways after Brexit demonstrates that the window of access is itself a structural determinant of outcome.

Five directions are proposed for future research. The most urgent is empirical testing of the Double Displacement concept against real data with actual participants across multiple migration corridors, to determine whether the analytically derived framework holds, requires revision, or generates unexpected findings. Longitudinal designs tracking late-career trajectories across AI adoption cycles would test whether the compounding dynamic intensifies as retirement approaches and AI deployment matures. Gender is the underexplored fourth intersecting axis: the study noted gendered dimensions of the resistant label finding in Theme 8, and future research should treat gender, migration status, age, and AI displacement as four interacting dimensions rather than three. Sector-specific investigation of the expropriation mechanism, particularly in healthcare and finance where training data dynamics are most clearly documented and regulatory implications most significant, warrants dedicated attention. Finally, quantitative testing of the buffer conditions identified in Themes 5 and 10 as predictive factors for displacement intensity would determine whether their explanatory power is sufficient to support intervention design.

The concept of Double Displacement proposed here is, at this stage, an analytically derived framework awaiting empirical test. What the findings establish is that the question it names is real, that the population it concerns is identifiable, and that the convergence it describes is sufficiently specific and sufficiently consequential to warrant the sustained research programme it has not yet received.

## Data Availability

The raw data supporting the conclusions of this article will be made available by the authors, without undue reservation.
